# Prognostic Significance of PSMD1 Expression in Patients with Gastric Cancer

**DOI:** 10.7150/jca.31543

**Published:** 2019-07-20

**Authors:** Wenjun Xiong, Wei Wang, Haipeng Huang, Yuming Jiang, Weihong Guo, Hao Liu, Jiang Yu, Yanfeng Hu, Jin Wan, Guoxin Li

**Affiliations:** 1 Department of General Surgery, Nanfang Hospital, Southern Medical University, Guangdong Provincial Engineering Technology Research Center of Minimally Invasive Surgery, Guangzhou, China; 2 Department of Gastrointestinal Surgery, Guangdong Provincial Hospital of Chinese Medicine, the Second Affiliated Hospital of Guangzhou University of Chinese Medicine, Guangzhou, China

**Keywords:** PSMD1, prognostic significance, gastric cancer

## Abstract

**Background**: PSMD1 has been considered to be involved in many human cancers, but its prognostic significance in gastric cancer (GC) has not been elucidated. The aim of this study was to evaluate the prognostic value of PSMD1 expression in tumor tissues of GC patients.

**Methods**: We retrospectively analyzed the expression of PSMD1 in 241 paraffin-embedded GC specimens of the training cohort by immunohistochemistry. The prognostic value of PSMD1 expression was assessed using Kaplan-Meier survival curves and multivariate COX regression models. PSMD1 expression and other GC-associated risk factors were used to generate two nomograms to evaluate prognosis, and the performance of the two nomograms was assessed with respect to its calibration, discrimination, and clinical usefulness. Further validation was performed using an independent cohort of 170 cases.

**Results**: High PSMD1 expression was significantly associated with decreased disease-free survival (DFS) and overall survival (OS) in GC patients. Furthermore, multivariate Cox proportional hazard analysis demonstrated that PSMD1 was an independent prognostic factor for DFS and OS. The two nomograms that were developed by integrating PSMD1 expression and the TNM staging system showed better prediction of DFS and OS than TNM staging system alone(C-index for training cohort: 0.708 (95% CI:0.670-0.746) and 0.712 (0.671-0.752), respectively; C-index for validation cohort: 0.704 (0.651-0.757) and 0.711 (0.656-0.767), respectively). Decision curve analysis demonstrated that the nomograms showed potential for clinical use.

**Conclusion**: Intratumoral PSMD1 expression is a novel independent predictor of DFS and OS in GC patients. In the future, large-scale prospective studies will be necessary to confirm our findings regarding its potential prognostic and therapeutic value for GC patients.

## Introduction

Gastric cancer (GC) is the most common cause of cancer deaths worldwide 1. Despite efforts to improve the survival of patients with GC, satisfactory outcomes have not been achieved. Currently, the prognostic models for GC are mainly based on the International Union Against Cancer (UICC) Tumor-Node-Metastasis (TNM) staging system. However, the outcomes for patients with a similar TNM stage are highly variable because of the inherent heterogeneity 2-5. Therefore, stratifying patients in the current TNM staging system based on the molecular factors that are involved in gastric carcinogenesis improves the prediction of the clinical outcomes 3.

Proteasomes are multisubunit proteases that mediate the degradation of ubiquitinated proteins 6. The 19S regulatory particle (19S-RP) of the 26S proteasome directs the ubiquitinated degradation substrates into the proteasome's catalytic 20S core particle (20S-CP) of the proteasome. PSMD1 (Rpn2 in yeast) is the largest subunit of 19S-RP. PSMD1is a key structural component of the 19S-RP, which acts as a docking site for other proteasomal subunits. Many tumor suppressor and oncogenic proteinsare regulated by ubiquitin-proteosome mediated protein degradation 7, 8. Therefore, it is conceivable that PSMD1 plays an important role in regulating carcinogenesis and cancer progression. Recent studies show that PSMD1 is upregulated in anaplastic thyroid carcinoma (ATC) and breast cancer tissues and shows potential as a novel therapeutic target 9, 10. Okumura et al. showed that PSMD1as a potential critical gene that may regulate cell proliferation and cell-cycle progression by mediating p53 protein degradation in breast cancer cells 10. However, the role and prognostic potential of PSMD1 in GC are not yet known.

In this study, through quantitative polymerase chain reaction (qPCR) and immunohistochemistry assays, we found that GC tissues showed high PSMD1 protein expression. We further demonstrated that closely associations existed between PSMD1 levels and clinicopathological factors. Therefore, we postulate that PSMD1 is a potential target for diagnosis and clinical treatment of GC. We also developed two predictive nomograms to assess the risk score for overall survival (OS) and disease-free survival (DFS) of GC patients by integrating parameters such as PSMD1 expression, tumor depth, lymph node metastasis and distant metastasis.

## Materials and Methods

### Patients and tissue specimens

Thirty-six fresh frozen GC and corresponding nontumoral gastric mucosa tissue samples were taken from patients with GC within 30 min after resection, and then snap-frozen in liquid nitrogen and stored at -80 ºC until use **(Table S1)**. The thirty-six patients were availability of hematoxylin and eosin slides with invasive tumor components, availability of complete clinicopathologic characteristics, no preoperative anticancer treatment, and more than 15 examined lymph nodes. Furthermore, we enrolled two independent panels of formalin-fixed paraffin-embedded (FFPE) specimens derived from 411 GC patients for this study. This included a training cohort of 241 patients with incident, primary, biopsy-confirmed GC, diagnosed from June 2006 to April 2008 at Nanfang Hospital of Southern Medical University (Guangzhou, China). Inclusion criteria were availability of hematoxylin and eosin slides with invasive tumor components, availability of follow-up data and clinicopathologic characteristics, no history of cancer treatment, more than 15 examined lymph nodes, and appropriate patient informed consent. We excluded patients if formalin-fixed paraffin-embedded (FFPE) tumor and normal samples from the initial diagnosis were unavailable or if they had received previous treatment with any anticancer therapy. Two independent pathologists reassessed all these samples. The internal validation cohort included an additional 170 patients, with the same criteria as above, diagnosed from May 2008 to May 2009 at Nanfang Hospital of Southern Medical University. TNM staging was reclassified according to the AJCC staging manual (seventh edition). Two independent pathologists reassessed all these samples. This study was approved by the research ethics committee at Nanfang Hospital of Southern Medical University and the need to obtain informed consent was waived because of the retrospective nature of the study.

### RNA extraction and quantitative PCR

Total RNA was extracted with TRIzol reagent (TaKaRa, Dalian China) and cDNA was synthesized from 5 ng of total RNA using the mRNA reverse transcription kit (TaKaRa, Dalian China). We performed real-time PCR to quantitate mRNA expression as described in the protocol supplied with SYBR Premix Ex Taq (Takara) using a LightCycler 480 v.1.5 system (Roche). PSMD1 gene expression were normalized to GAPDH gene expression (internal control) using the 2-ΔΔCt method 11, 12.

### Immunohistochemistry (IHC)

FFPE samples were subjected to IHC as previously described 13-15. The samples were cut into 4 µm-thick sections, de-waxed in xylene, and rehydrated in decreasing concentrations of ethanol. Prior to staining, sections were blocked with endogenous peroxidase (prepared in 1% H_2_O_2_/methanol solution) for 10 min and then microwaved for 30 min in 10 mM citrate buffer, pH6.0. Then, the sections were blocked using 10% normal rabbit serum for 30 min. The slides were incubated overnight withanti-PSMD1 antibody (1:200 dilution; ab140677; Abcam, Cambridge, MA) at 4℃, followed by incubation with an amplification system with a labeledpolymer/HRP (EnVision™, DakoCytomation, Denmark) for 30 min. The sections were developed with 0.05% 3, 3´-diaminobenzidine tetrahydrochloride (DAB) and counterstained with modified Harris hematoxylin.

### Evaluation of IHC staining

Two experienced pathologists who were blinded to the clinical parameters and outcomes for each patient independently reviewed the IHC stained sections. Each section was scored by randomly selecting and examining 10 fields in the tumor region with a high-power microscope. Their results were in complete agreement in approximately 90% of the cases. A third pathologist was consulted when different opinions arose between the two primary pathologists. If the third pathologist agreed with one of them, then that value was selected. If the conclusion by the third pathologist was completely different, then the three of them would work collaboratively to find a common answer. PSMD1 expression in each specimen was evaluated by determining a semi-quantitative H score, which was calculated by multiplying the result of a 4-stepscale (0 = negative, 0.5 = weak staining, 1 = moderate staining, 1.5 = strong staining) and the fraction of positively stained cells that ranged from 0 to 100%^16^, ^17^. The H-scores for PSMD1 staining were dichotomized at the median to classify GC patients into high and low PSMD1 expression groups.

### Construction of the Nomograms

In the training cohort, survival curves for different variables were generated using the Kaplan-Meier estimates and were compared using the log-rank test. Variables that achieved statistical significance with a *P*< 0.05 were subjected to multivariable analyses using the Cox regression model. Statistical analysis to identify independent prognostic factors was performed by using the SPSS 19.0 software for Windows (SPSS, Chicago, IL). Based on the results of the multivariable analysis, two nomograms were established using the survival and rms package of the R 3.4.0 software (http://www.r-project.org). Backward step-wise selection was determined by the likelihood ratio test using Akaike's information criterion as the stopping rule 18.

### Validation and Calibration of the Nomograms

The performance of the two nomograms for predicting survival outcomes was tested in the validation cohort by calculating the concordance index (C-index) 19. The value of the C-index ranged from 0.5 to 1.0, with 0.5 indicating a random chance and 1.0 indicating a perfect clear-cut ability to correctly determine the survival outcome with the model. The nomograms were calibrated for 1-, 3-, and 5-year DFS and OS by comparing predicted survival with observed survival after bias correction.

### Clinical Use

Decision curve analysis was performed to determine the clinical usefulness of the nomograms by quantifying the net benefits at different threshold probabilities 20, 21.

### Risk Group Stratification Based on the Nomogram

The composite scoring of the nomograms was divided into three risk groups using X-tile 22, which accurately discriminated patients with good, intermediate, and poor prognosis.

### Statistical analysis

We analyzed the statistical data for two groups by using the *t* test for continuous variables and χ² test for categorical variables. The DFS and OS were defined as the number of months from the date of surgery to the date of regional recurrence or distant metastasis (for DFS) and death or final clinical follow-up (for OS). The Kaplan-Meier method and the log-rank test were used to estimate DFS and OS. Multivariate Cox proportional hazards regression analysis was performed for all variables that were significant in the univariate analysis. The statistical tests were performed using the R (version 3.4.0)and SPSS (version 19.0) software packages. All statistical tests were two-sided, and *P*< 0.05 was regarded as statistically significant.

## Results

### Overexpression of PSMD1 in human GC

**Figure 1:**
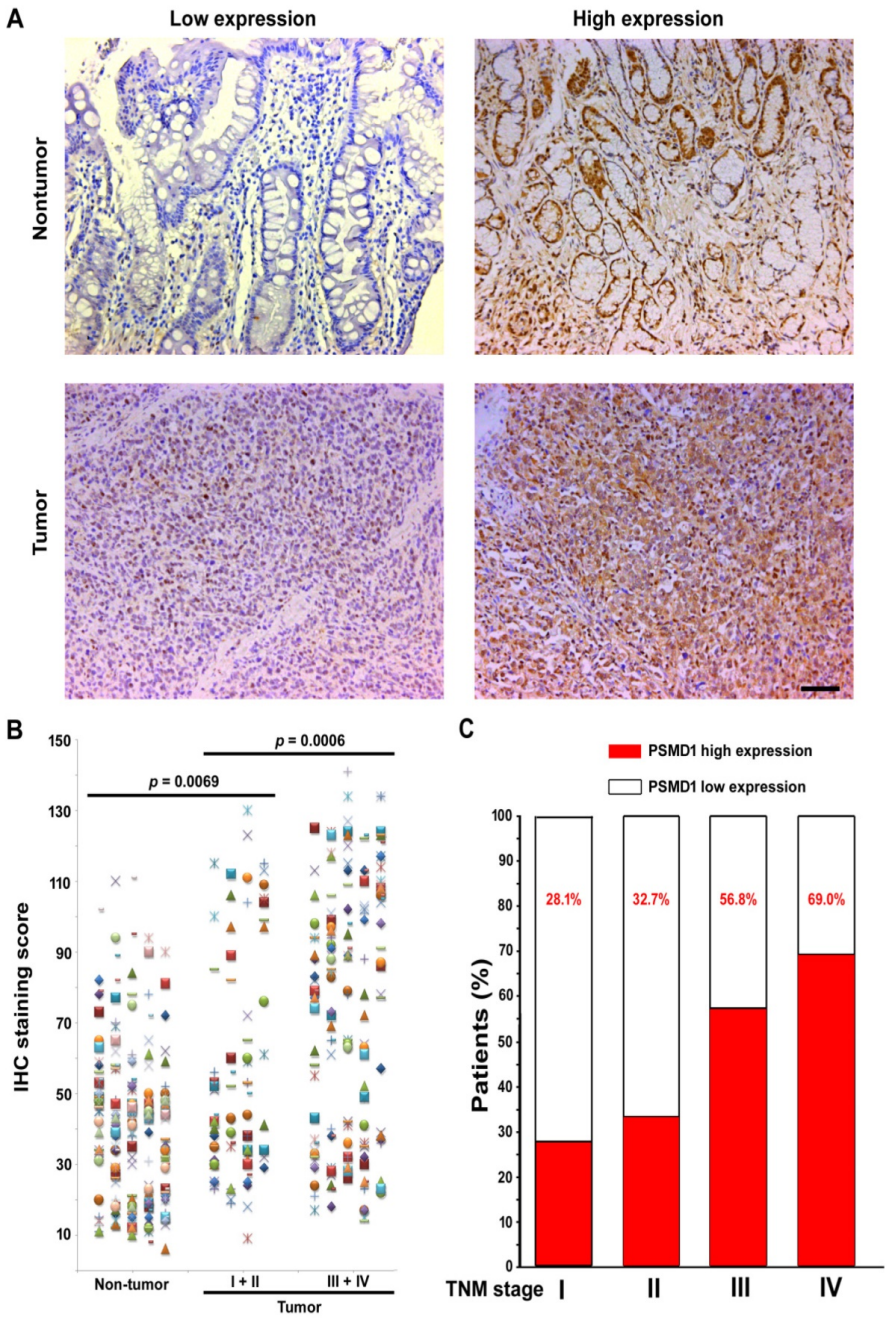
PSMD1 expression in GC tissues. (A) Representative IHC images of PSMD1 expression in nontumor and tumor tissues of GC patients. Left panel: low expression; right panel: high expression. (B) Scatter plots for IHC staining score in unpaired nontumor (n = 241) and tumor (n = 241) tissues from the training cohort. P value was determined using the nonparametric Mann-Whitney test. c Increased percentage of GC patients belonging to TNM stages I-IV show higher intratumoral PSMD1 expression suggesting its association with disease progression (data from the training and validation cohorts). Scale bar, 100 µm.

To elucidate the role of PSMD1 in the initiation and progression of GC, we first analyzed its expression by real-time PCR in 36 GC biopsies and matched adjacent nontumoral tissues at the mRNA level. mRNA level analysis of PSMD1 expression in matched nontumoral and tumor tissues showed that PSMD1 was upregulated in the majority of GC tissues compared to their nontumoral counterparts (25/36; 69.4%) (**Fig. S1**), and PSMD1 protein levels were also significantly higher in tumor tissues (**Fig. S2**). Furthermore, compared with the nontumoral PSMD1 density, intratumoral PSMD1 expression was also higher (*P* < 0.0001; **Fig. 1A-B**), as shown by IHC findings. **Table 1** lists the detailed clinicopathological characteristics of the GC patients in the training and validation cohorts. The expression of PSMD1 was much higher in advanced stage GC [stages I-II (n =80) vs. stages III-IV (n = 161), *P* = 0.0006]. Furthermore, the percentage of patients with high intratumoral PSMD1 expression increased moderately from TNM stages I to IV, suggesting its association with disease progression (**Fig. 1C, Table 1**).

### High expression of PSMD1 is associated with poor clinical outcomes

**Figure 2:**
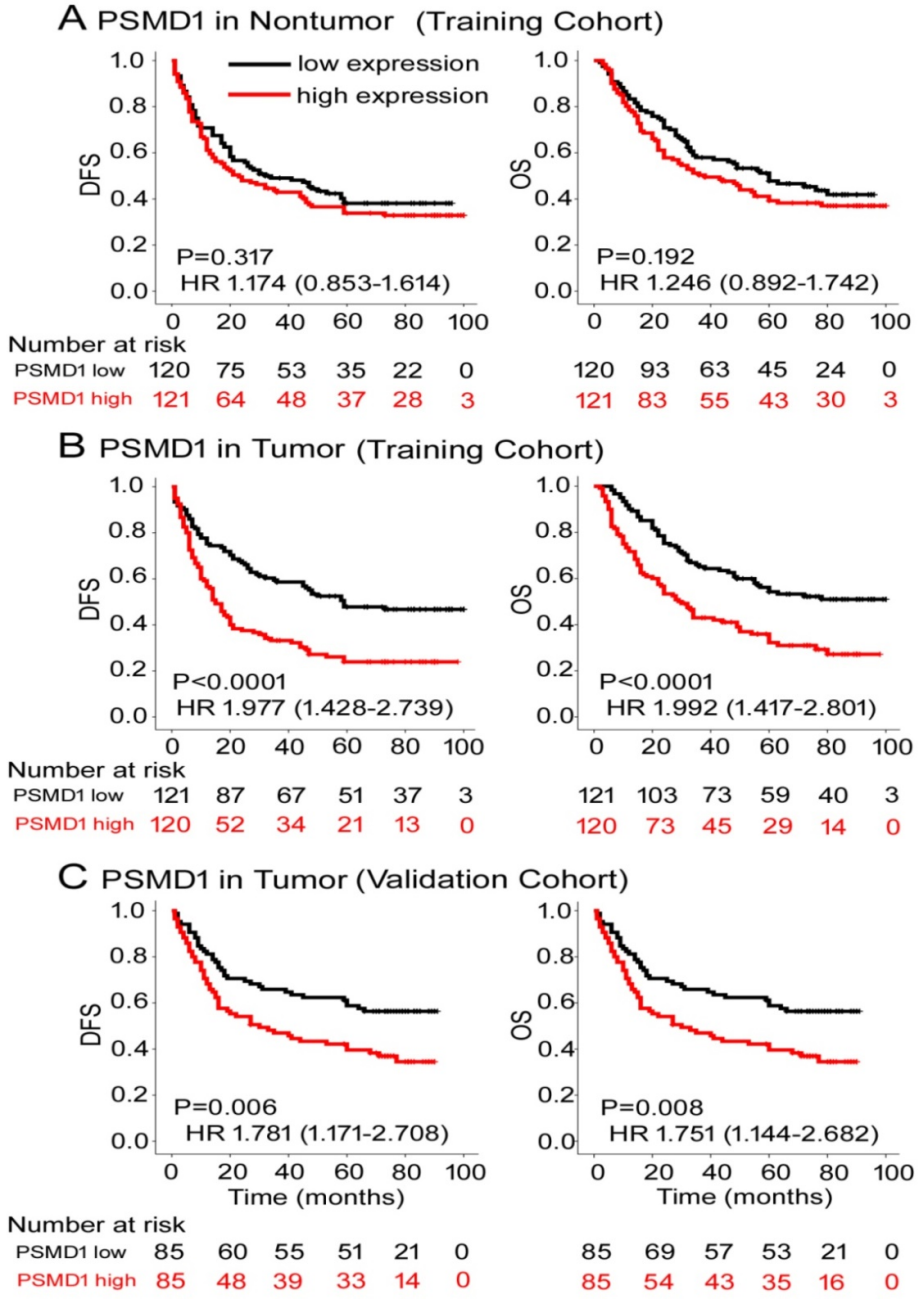
Kaplan-Meier survival analyses of disease-free survival (DFS) and overall survival (OS) in the training and validation cohorts of GC patients classified according to PSMD1 expression in **(A)** nontumor and tumor **(B, C)** tissues.

To determine the prognostic value of PSMD1 expression in GC patients, Kaplan-Meier survival analysis was performed to analyze DFS and OS based on PSMD1 expression. In the training cohort, PSMD1 expression in non-tumor tissue was not associated with DFS and OS (**Fig. 2A**). However, patients with high intratumoral PSMD1 expression were associated with lower 5-year DFS and OS than patients with low intratumoral PSMD1 expression (5-year DFS: 17.5% vs. 42.1%; hazard ratio (HR) =1.977 (1.428-2.739); 5-year OS: 24.2% vs. 48.9%; HR=1.992 (1.417-2.801); all P < 0.001; **Fig. 2B**). We obtained similar results in the validation cohort (**Fig. 2C**), which confirmed the prognostic significance of PSMD1 expression in different populations of GC patients. Multivariate analysis showed that PSMD1 was an independent prognostic factor for predicting DFS and OS in GC patients (**Table 2 and S2-3**). When stratified by clinicopathological risk factors, PSMD1 remained a clinically relevant prognostic marker (**Fig. S3-4**).

### Development and validation of nomograms for predicting prognosis of GC patients

**Figure 3:**
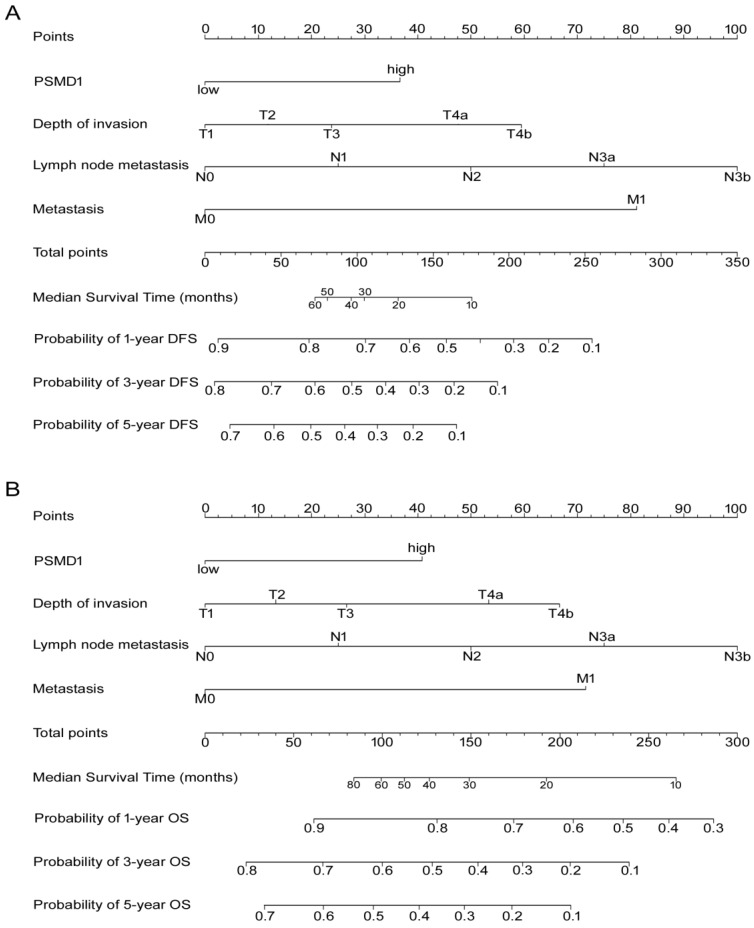
Nomogram for predicting DFS and OS. The grade of the patient is indicated on the grade axis and a straight line is drawn upwards to the point's axis to determine the number of points received by the patient towards survival for his/her grade. This process is repeated for the other axes to determine the sum of the points received for each predictor. A straight line is drawn down to the survival-probability axis to determine the probability of the patient surviving gastric cancer.

**Figure 4:**
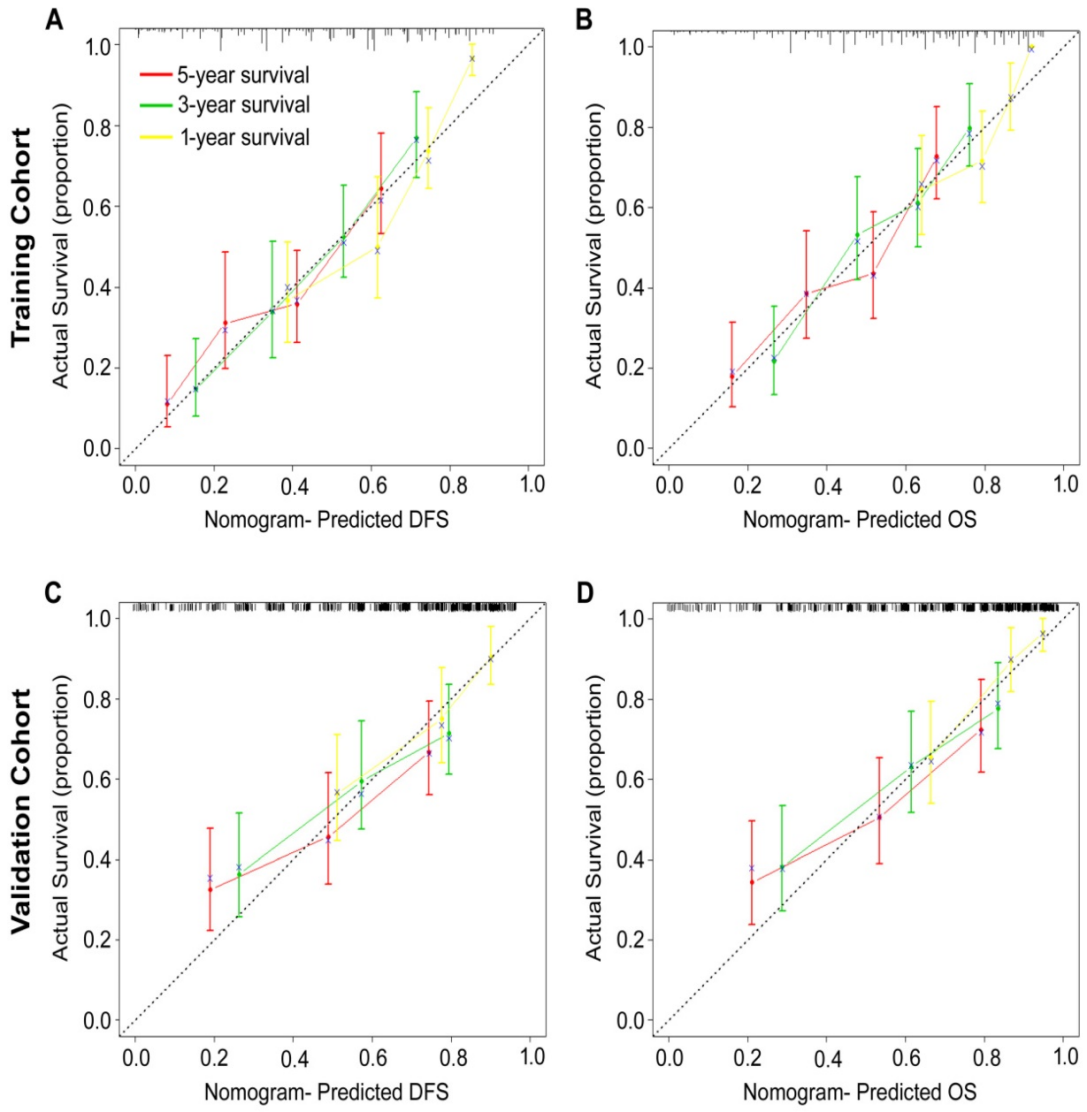
Calibration curves for the nomogram-predicted probability of 1-, 3- and 5-year **(A, C)** DFS and **(B, D)** OS of GC patients in the training and validation cohorts. The nomogram-predicted OS and DFS values are plotted on the x-axis, and the actual OS and DFS values are plotted on the y-axis. The dotted line represents an ideal nomogram, and the solid blue line represents the current nomogram. The vertical bars represent 95% CIs, and the ×'s represent bootstrap-corrected estimates.

**Figure 5:**
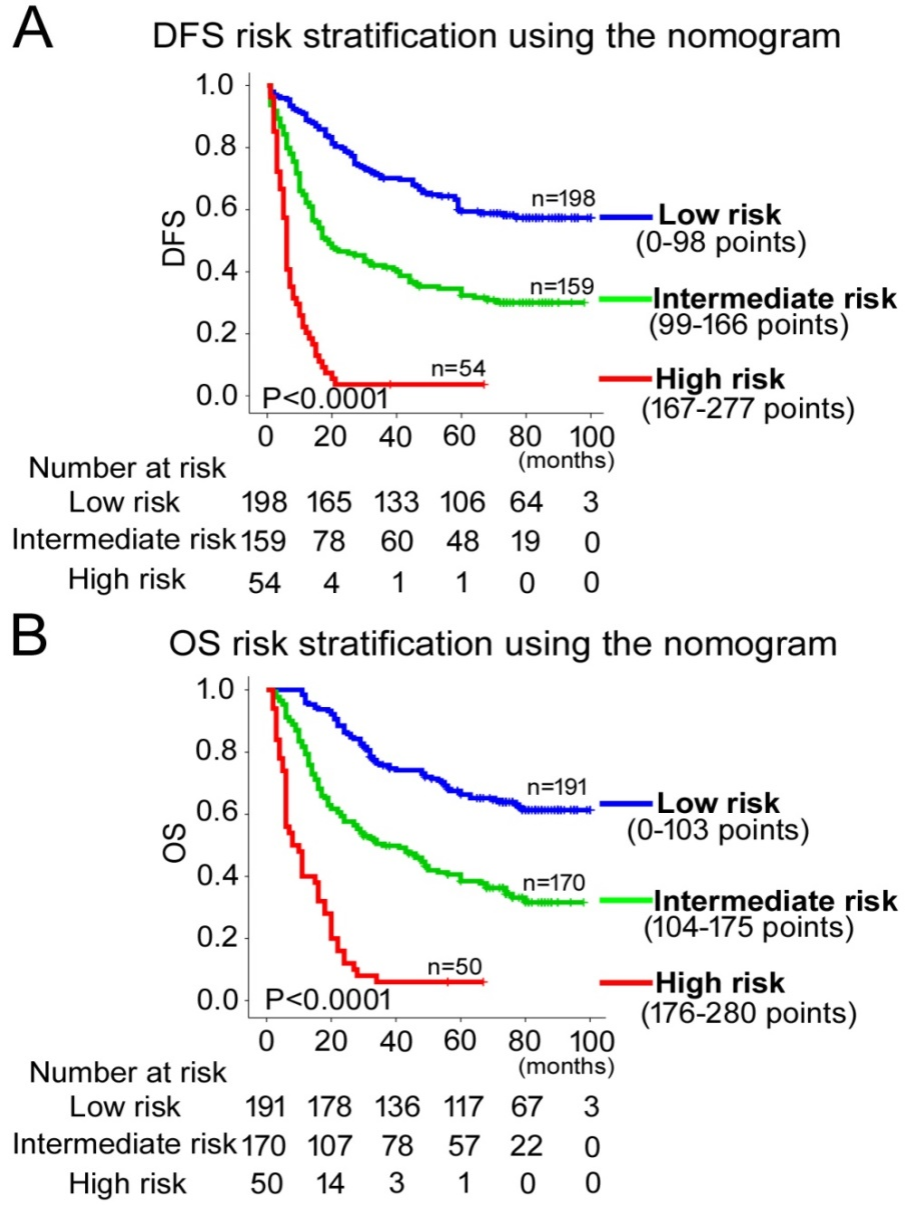
Kaplan-Meier survival analyses of OS and DFS for the three risk groups. The entire patient population was divided into 3 subgroups based on the total number of points obtained in the **(A)** DFS and **(B)** OS nomograms.

According to all significant independent risk factors for survival of GC patients, we established two nomograms using the multivariate Cox regression model to predict DFS and OS of GC patients (**Fig. 3**). The nomograms can be interpreted by adding the points assigned to each significant variable that are indicated at the top of the scale. The total points can then be used to predict 1-, 3- and 5-year DFS and OS for a patient in the lowest scale 23. In the training cohort, the C-indexes for DFS and OS prediction were 0.708 (95% CI: 0.670-0.746) and 0.712 (95% CI: 0.671-0.752), respectively. Recalibration was not required because the calibration curves for the two nomograms (**Fig. 4A-B**) did not deviate from the reference line. In the validation cohort, the C-indexes for DFS and OS prediction were 0.704 (0.651-0.757) and 0.711 (0.656-0.767), respectively. The calibration curves yielded good agreement between the predicted and observed outcomes for both DFS and OS (**Fig. 4C-D**). Furthermore, the performances of the nomograms for DFS and OS in both the training and validation cohorts were superior relative to the TNM staging system alone **(Fig. S5**). The C-indexes of TNM stage for DFS and OS prediction were 0.681 (0.648-0.715) and 0.678 (0.640-0.716) in the training cohort, and 0.660 (0.610-0.709) and 0.661 (0.610-0.712) in the validation cohort, respectively. Using the X-tile, the composite scoring was divided into three risk groups that accurately discriminated between patients with good, intermediate, and poor prognosis (**Fig. 5 and Fig. S6**).

### Clinical Use

**Figure 6:**
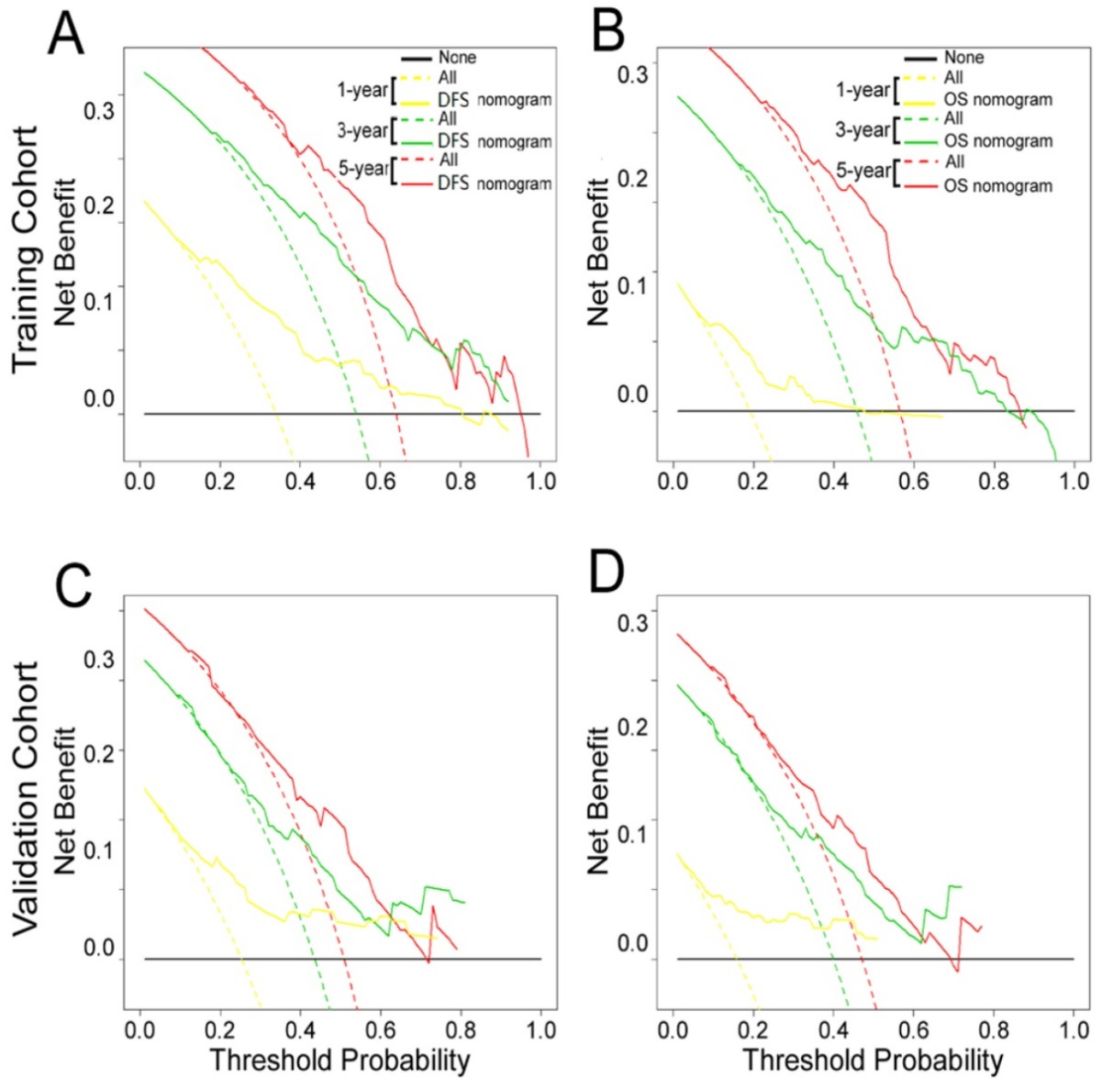
Decision curve analysis of the **(A, C)** DFS and **(B, D)** OS nomograms for the training and validation cohorts. The y-axis represents net benefit and the x-axis represents threshold probability. The solid lines (yellow, blue and red) represent the nomograms. The dotted lines (yellow, blue and red) represent the assumption that all patients survive for1-, 3-, or 5-years, respectively. The thin black line represents the assumption that none of the patients survive for 1-, 3-, or 5-years.The net benefit was calculated by subtracting the number of false positive patients from the number of true positive patients, taking into consideration the relative harm of foregoing treatment compared to the negative consequences of unnecessary treatment.^15,16^ Relative harm was calculated by [pt/(1 -pt)]; “pt” represents the threshold probability where the expected benefit of treatment equals the expected benefit of avoiding treatment. The equation ([a - c]/[b - d] = [1 - pt]/pt) shows how a patient weighs the relative harms of false-positive and false-negative results at the time when he/she opts for treatment; 'a - c' represents the harm from a false-negative result; 'b - d' represents the harm from a false-positive result; And a, b, c and d represent the values for true positive, false positive, false negative, and true negative results, respectively.^ 15,16^ Training cohort: **(A, B)**. Validation cohort: **(C, D)**.

The decision curve analysis for the two nomograms is shown in **Fig. 6.** The decision curve showed that if the threshold probability of a patient or doctor was > 10%, predicting the 1-, 3-, 5-year DFS and OS was more accurate using the two nomograms than either the treat-all-patients or treat-none schemes.

## Discussion

To our knowledge, this is the first study to analyze the potential prognostic value of PSMD1 in GC. We observed variable expression of PSMD1 in GC tissues, and positive correlation of PSMD1 expression with TNM stage in tumor tissues. Moreover, high expression of PSMD1 indicated a more aggressive tumor phenotype and was associated with poor DFS and OS.

The 26S proteasome is a multicatalytic proteinase complex that is composed two complexes (20S core and 19S regulator). The PSMD1 gene encodes the largest non-ATPase subunit of the 19S regulator lid, which is responsible for substrate recognition and binding. Ryu et al. showed that PSMD1 SUMOylation controls proteasome composition and function, thereby providing a mechanism for regulation of ubiquitin-mediated protein degradation through the SUMO pathway 24. Jonker et al. revealed that PSMD1was significantly upregulated in ATC and showed potential as a therapeutic target 13. Deng et al. showed high PSMD1 expression (>3-fold) in breast cancer tissues compared to adjacent normal tissues 25. Wang et al. used cDNA microarray analysis to compare gene expression profiles of acute promyelocytic leukemia cell line NB4 before and after (12 h) regular treatment and demonstrated high PSMD1 expression in the untreated cell line; this suggested a critical role for PSMD1 in the apoptosis and partial differentiation of NB4 cells 26. In addition, a group of genes encoding proteasomal subunits (PSMA1, PMSE1, PSMD1 and PMSD8) that showed hypomethylated promoters were upregulated in pre-eclampsia 27. Okumura et al. showed that PSMD1 was associated with poor prognosis of breast cancer patients based on the analyses of a clinical dataset (*http://kmplot.com*). Lagadecet al. showed that weak PSMD1 expression in the head and neck cancer cells predicts unfavorable outcomes post-radiotherapy 28. In the present study, we initially showed higher PSMD1expression in GC tissues than in the surrounding nontumor mucosal tissues by performing qRT-PCR fresh-frozen GC tissues and IHC in FFPE GC specimens. Moreover, high PSMD1 expression was associated with poor prognosis of GC patients.

The proteasome inhibitors, carfilzomib and bortezomib, inhibit ATC tumor growth, both in vitro and in vivo 7, 8. Bortezomib also induced redifferentiation of ATC cells and increased their iodine uptake. The activity of the ubiquitin-proteasome system is obviously enhanced in many types of human cancer, and its selective inhibition is a potential target for treating human cancers 8, 25. The knockdown of proteasome 26S subunit PSMD1 markedly reduced the proliferation of4-hydroxytamoxifen (OHT) resistant MCF-7 (OHTR) breast cancer cells 10. Notably, the knockdown of PSMD1 resulted in accumulation of the p53 protein and subsequent cell cycle arrest. Furthermore, in accordance with p53accumulation, PSMD1 silencing also upregulated p21 and SFN, the target genes of p53. Therefore, PSMD1 may play a role in the development of tamoxifen resistance in breast cancer cells. Our finding provides a new insight for the mechanism underlying the therapeutic resistance of gastric cancer and identifies PSMD1 as a potential prognostic and therapeutic target.

Although several prognostic biomarkers have recently been identified for gastric cancer recently 14, 17, TNM staging remains the most commonly used system to predict survival for GC patients. However, GC patients within the same TNM stage demonstrate different cellular, genetic, and clinicopathological characteristics. Moreover, their survival is highly variable, and TNM staging may not be sufficient to accurately predict GC patients survival 2. Nomograms have been developed to evaluate a large number of significant clinicopathological factors to improve prognostic prediction of individual patients and to provide a more individualized staging system. Improved prediction of individual outcomes would be useful for counseling patients, personalized treatment, and scheduling patient follow-ups 29. In our study, PSMD1 expression demonstrates sufficient discriminatory power in most subgroups of different clinicopathological types based on Kaplan-Meier survival curves and univariate COX stratification analysis (**Fig. S1-2**). Furthermore, multivariate COX regression analysis confirmed the independent prognostic value of PSMD1 expression, which could be integrated with the TNM staging system in the nomogram (**Table 2**). Nomograms were validated using calibration plots and the C-index. The nomograms performed well with good discrimination and calibration, and the C-index for DFS and OS was satisfactory. AUC and C-index values indicated that the nomogram performed better than the TNM stage alone (**Fig. S3**). However, to overcome the limitations of our study as a result of its retrospective design and the relatively small size of the patient population, we propose that a large-scale, multicenter, prospective study is needed to validate our results.

In conclusion, our study demonstrates PSMD1overexpression in GC tissues. GC patients with high intratumoral PSMD1 expression exhibit poorer survival than patients with low intratumoral PSMD1 expression. Our results demonstrate that PSMD1 expression is an independent predictor of GC patient outcomes. Integrated analysis of PSMD1 expression with TNM staging system provides a better prognostic model for GC patients and shows potential to aid both clinicians and patients in regard to counseling, individualized adjuvant treatment decision-making and follow-up scheduling.

## Supplementary Material

Supplementary figures and tables.

## Figures and Tables

**Table 1:** Clinical characteristics of patients according to PSMD1 in the training and validation cohorts.

Variables	Training cohort (n = 241)					Validation cohort (n =170)			
	N		low PSMD1(%)	high PSMD1(%)	p value	N	low PSMD1(%)	high PSMD1(%)	p value
**Gender**					0.141				0.219
Female	68		29(42.6%)	39(57.4%)		80	44(55.0%)	36(45.0%)	
Male	173		92(53.2%)	81(46.8%)		90	41(45.6%)	49(54.4%)	
**Age(years)**					0.941				0.442
<60	138		69(50.0%)	69(50.0%)		91	48(52.7%)	43(47.3%)	
≧60	103		52(50.5%)	51(49.5%)		79	37(46.8%)	42(53.2%)	
**Tumor location**					0.125				0.003
Higher	49		32(65.3%)	17(34.7%)		34	13(38.3%)	21(61.8%)	
Moderate	39		19(48.7%)	20(51.3%)		32	12(37.5%)	20(62.5%)	
Lower	119		54(45.4%)	65(54.6%)		84	54(64.3%)	30(35.7%)	
Whole	34		16(47.1%)	18(52.9%)		20	6(30.0%)	14(70.0%)	
**Size**					0.108				0.039
<4	123		68(55.3%)	55(44.7%)		63	38(60.3%)	25(39.7%)	
≧ 4	118		53(44.9%)	65(55.1%)		107	47(43.9%)	60(56.1%)	
**Differentiation status**					0.254				0.02
Well	44		27(61.4%)	17(38.6%)		20	5(25.0%)	15(75.0%)	
Moderate	80		39(48.8%)	41(51.3%)		55	25(45.5%)	30(54.5%)	
Poor and undifferentiated	117		55(47.0%)	62(53.0%)		95	55(57.9%)	40(42.1%)	
**CEA**					0.015				0.002
Elevated	73		28(38.4%)	45(61.6%)		46	14(30.4%)	32(69.6%)	
Normal	168		93(55.4%)	75(44.6%)		124	71(57.3%)	53(42.7%)	
**CA199**					0.046				0.007
Elevated	74		30(40.5%)	44(59.5%)		41	13(31.7%)	28(68.3%)	
Normal	167		91(54.5%)	76(45.5%)		129	72(55.8%)	57(44.2%)	
**Depth of invasion**					0.007				0.038
T1	14		11(78.6%)	3(21.4%)		20	15(75.0%)	5(25.0%)	
T2	36		25(69.4%)	11(30.6%)		16	9(56.3%)	7(43.7%)	
T3	20		11(55.0%)	9(45.0%)		6	5(83.3%)	1(16.7%)	
T4a	131		59(45.0%)	72(55.0%)		91	41(45.1%)	50(54.9%)	
T4b	40		15(37.5%)	25(62.5%)		37	15(40.5%)	22(59.5%)	
**Lymph node metastasis**					0.003				0.497
N0		53	38(71.7%)	15(28.3%)		41	23(56.1%)	18(43.9%)	
N1		60	30(50.0%)	30(50.0%)		45	19(42.2%)	26(57.8%)	
N2		69	25(36.2%)	44(63.8%)		43	20(46.5%)	23(53.5%)	
N3a		39	20(51.3%)	19(48.7%)		24	12(50.0%)	12(50.0%)	
N3b		20	8(40.0%)	12(60.0%)		17	11(64.7%)	6(35.3%)	
**Metastasis**					0.036				0.115
M0		215	113(52.6%)	102(47.4%)		154	80(51.9%)	74(48.1%)	
M1		26	8(30.8%)	18(69.2%)		16	5(31.3%)	11(68.8%)	
**TNM stage**					<0.0001				<0.0001
I		19	14(73.7%)	5(26.3%)		32	23(71.9%)	9(28.1%)	
II		59	42(71.2%)	17(28.8%)		101	68(67.3%)	33(32.7%)	
III		137	57(41.6%)	80(58.4%)		236	102(43.2%)	134(56.8%)	
IV		26	8(30.8%)	18(69.2%)		42	13(31.0%)	29(69.0%)	
									

**Table 2:** Multivariable Cox regression analysis of disease-free survival and overall survival.

Variables	Disease-free survival			Overall survival	
	HR (95% CI)	p value		HR (95% CI)	p value
**PSMD1 (high vs. low)**	**1.603 (1.131-2.271)**	**0.008**		**1.584 (1.096-2.289)**	**0.014**
Depth of invasion		<0.001			0.003
T1	Reference			Reference	
T2	2.172(0.815-5.785)	0.121		5.172(1.203-22.23)	0.027
T3	0.907 (0.274-3.006)	0.874		2.187 (0.437-10.94)	0.341
T4a	1.811 (0.719-4.559)	0.208		4.255 (1.029-17.59)	0.046
T4b	4.166 (1.573-11.034)	0.004		7.048 (1.645-30.19)	0.009
Lymph node metastasis		0.002			0.036
N0	Reference			Reference	
N1	1.559 (0.895-2.716)	0.117		1.558 (0.865-2.805)	0.140
N2	1.933 (1.130-3.306)	0.016		1.743 (0.982-3.094)	0.058
N3a	2.622 (1.470-4.678)	0.001		2.293 (1.240-4.241)	0.008
N3b	3.282 (1.698-6.343)	0.0004		2.796 (1.373-5.694)	0.005
Metastasis (M1 vs. M0)	2.537 (1.562-4.121)	0.0002		2.028 (1.198-3.434)	0.008
