# Supplementary material for: Prognostic Significance of PSMD1 Expression in Patients with Gastric Cancer
Source: J Cancer. 2019 Jul 20;10(18):4357–67. doi: 10.7150/jca.31543 (PMC6691719; doi:10.7150/jca.31543)
Supplement: Supplementary file 1 — Supplementary figures and tables. [file jcav10p4357s1.pdf]

SUPPLEMENTARY MATERIALS

Supplementary materials include five supplementary Figurers and two supplementary tables.

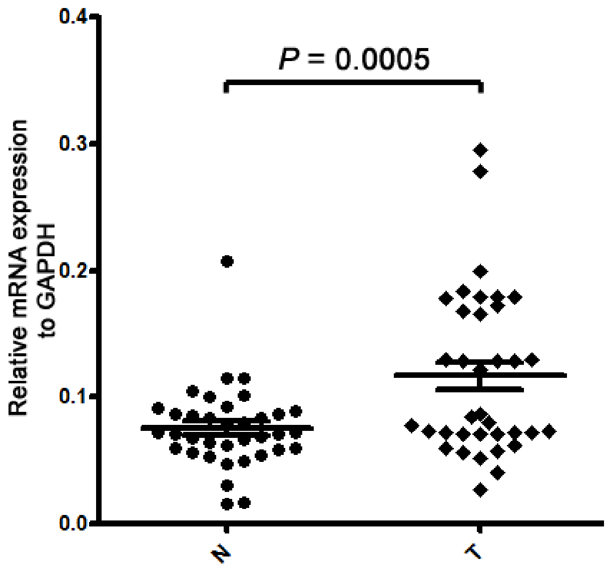

Figure. S1. PSMD1 gene expression in 36 gastric cancer biopsies and their matched adjacent nontumoral tissues detected by qPCR. Data were normalized to the mean Ct values of housekeeping gene GAPDH.

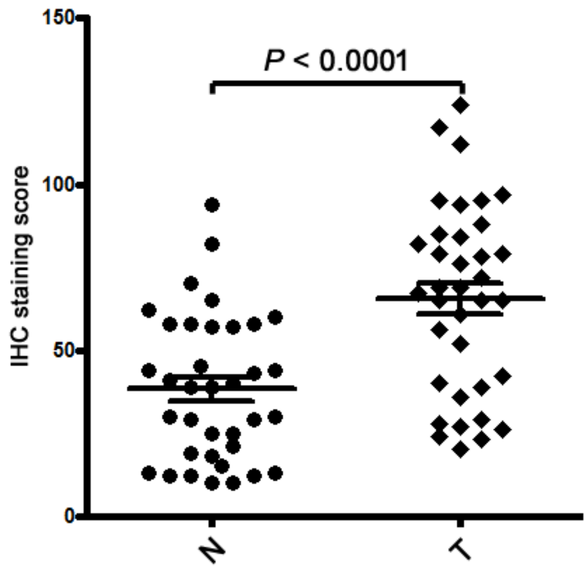

Figure. S2. Scatter plots for IHC staining score of PSMD1 in 36 paired nontumor and tumor tissues from the training cohort. N: nontumor; T: tumor. IHC: Immunohistochemistry.

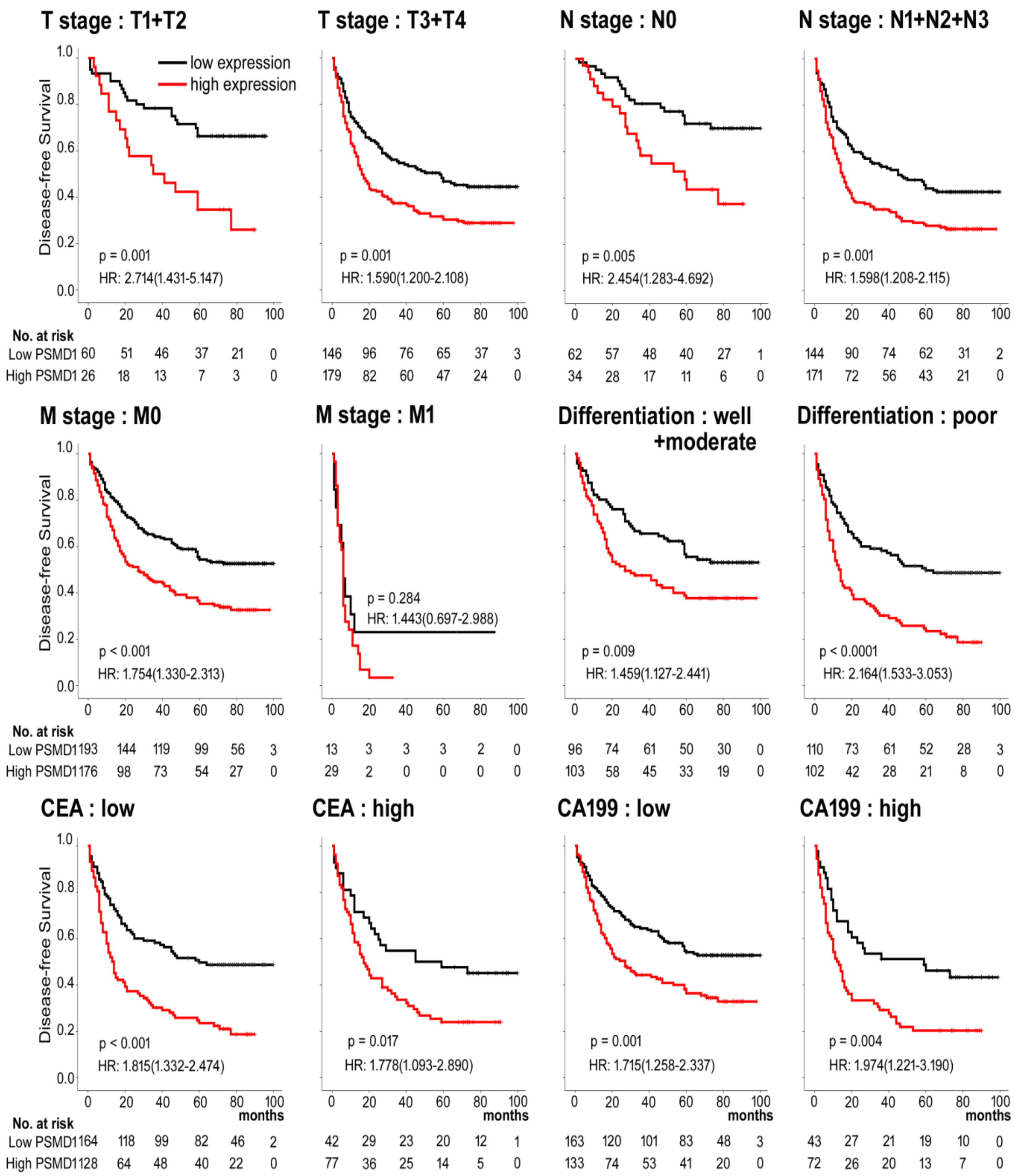

Figure. S3. Kaplan-Meier survival curve analysis of DFS for all 411 patients with gastric cancer according to the PSMD1 expression stratified by clinicopathological risk factors. *P*-values were calculated by the log-rank test.

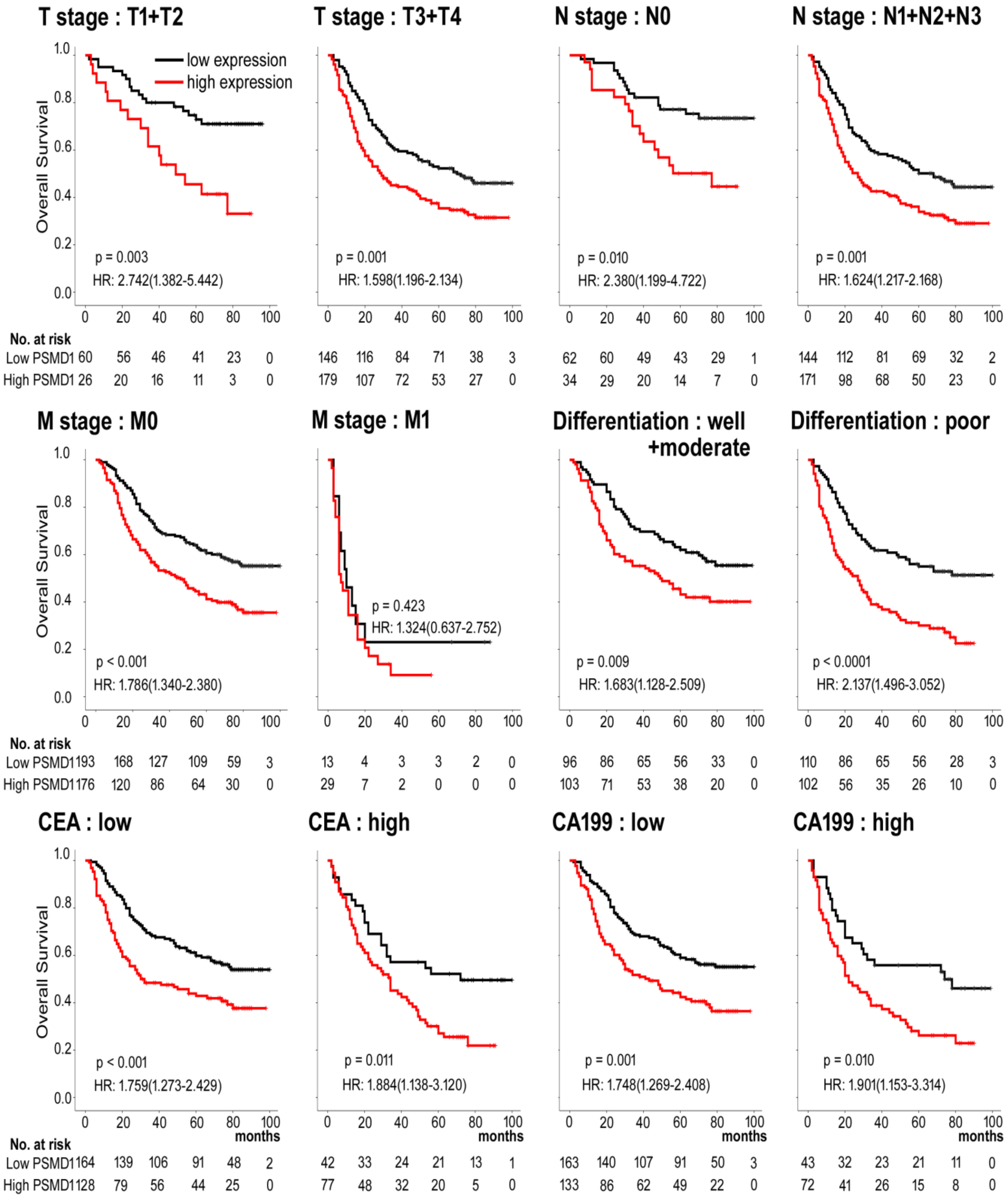

Figure. S4. Kaplan-Meier survival curve analysis of OS for all 411 patients with gastric cancer according to the PSMD1 expression stratified by clinicopathological risk factors. *P*-values were calculated by the log-rank test.

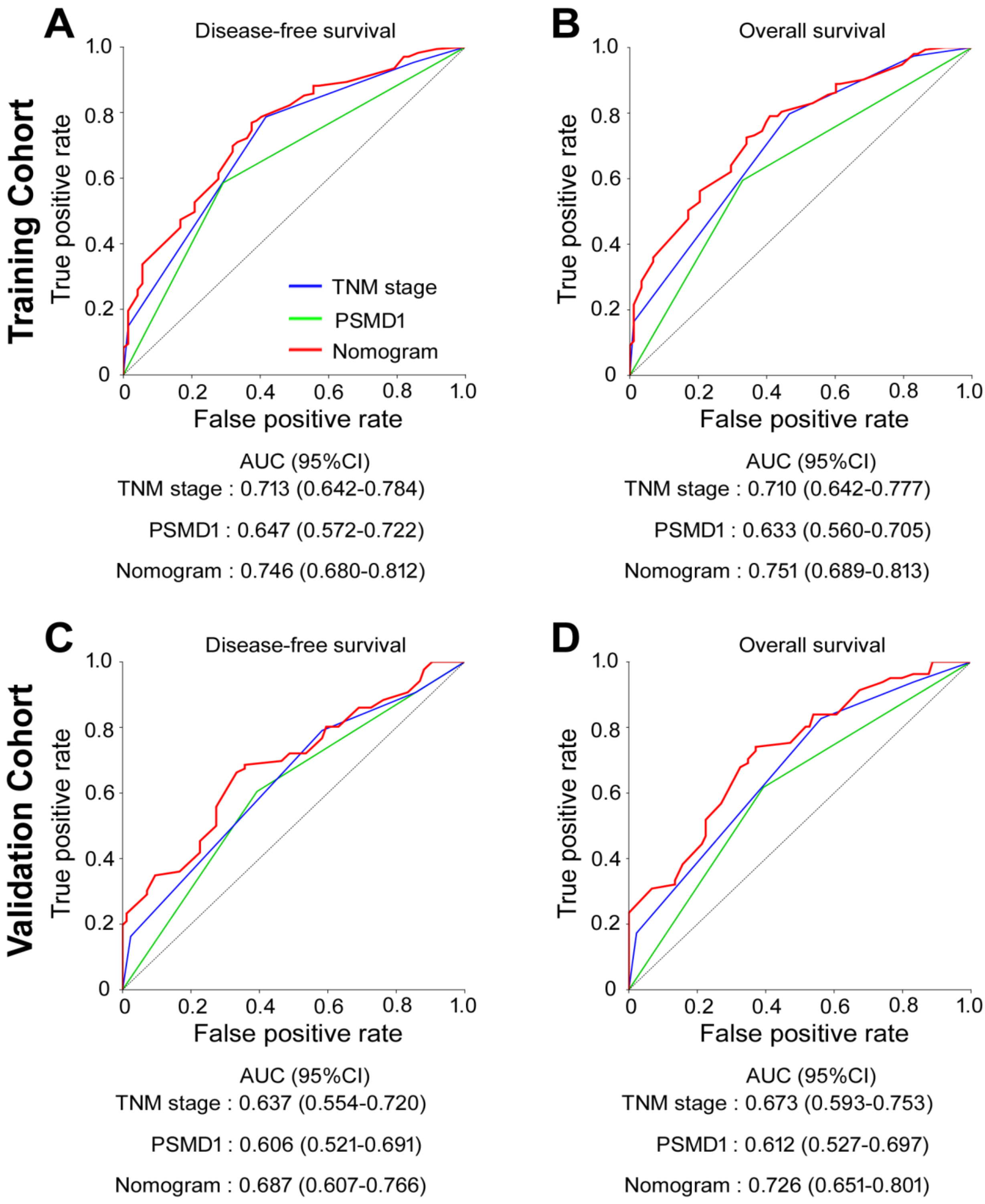

Figure. S5. Extended model for prediction of DFS and OS in patients with GC based on PSMD1 expression. ROC analyses of sensitivity and specificity for prediction of **a, b** DFS and **c, d** OS by the combined model (TNM stage and PSMD1 expression combination), TNM stage model, andPSMD1 expression model.

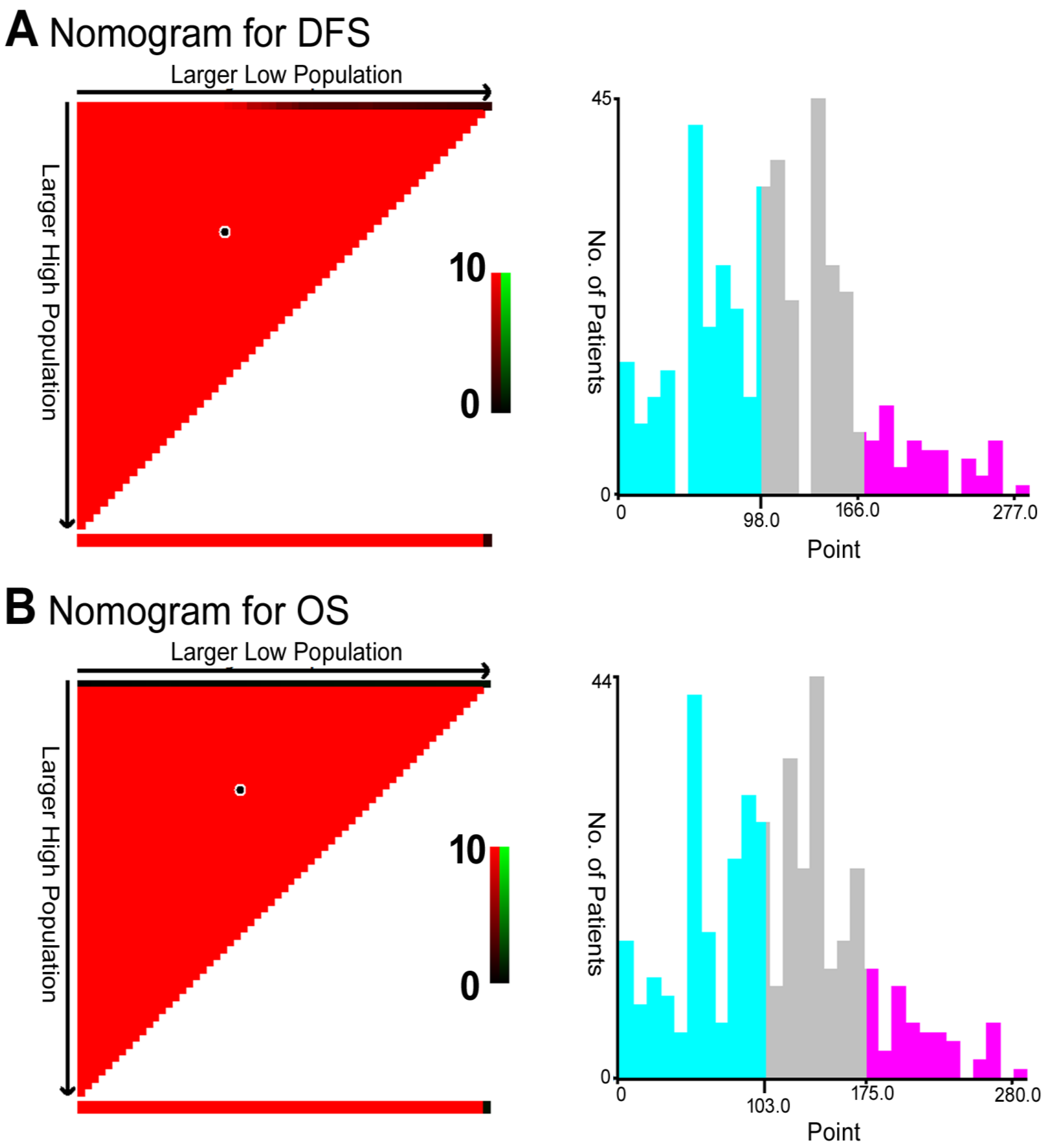

Figure. S6. X-tile plots of the DFS and OS nomograms. Color intensity of the plot represents low (dark, black) to high (bright, red, or green) strength of association at each division. Red represents the inverse association between the PSMD1 expression and survival (DFS or OS), whereas green represents direct association. **a** DFS nomogram, **b** OS nomogram.

Table S1. Clinical characteristics of the 36 patients with gastric cancer.

**Variables**	N	%
**Gender**		
Female	9	25.0%
Male	27	75.0%
**Age(years)**		
＜60	21	58.3%
≧60	15	41.7%
**Tumor location**		
Higher	12	33.3%
Moderate	4	11.1%
Lower	15	41.7%
Whole	5	13.9%
**Size**		
＜4 cm	23	63.9%
≧ 4 cm	13	36.1%
**Differentiation status**		
Well	7	19.4%
Moderate	14	38.9%
Poor and undifferentiated	15	41.7%
**CEA**		
Normal	25	69.4%
Elevated	11	30.6%
**CA199**		
Normal	26	72.2%
Elevated	10	27.8%
**Depth of invasion**		
T1	3	8.3%
T2	6	16.7%
T3	5	13.9%
T4a	19	52.8%

T4b	3	8.3%
**Lymph node metastasis**		
N0	14	38.9%
N1	10	27.8%
N2	7	19.4%
N3a	4	11.1%
N3b	1	2.8%
**Metastasis**		
M0	34	94.4%
M1	2	5.6%
**TNM stage**		
I	5	13.9%
II	13	36.1%
III	16	44.4%
IV	2	5.6%

Table S2: Univariate association of PSMD1, clinicopathological characteristics with disease-free survival and overall survival in the training cohort.

Variables	Disease-free survival		Overall survival	
	HR (95%CI)	p value	HR (95%CI)	p value
Age(years) (≥60 vs. <60)	1.055 (0.766-1.452)	0.744	1.194 (0.854-1.668)	0.300
Gender (female vs. male)	1.105 (0.774-1.577)	0.583	1.146 (0.786-1.671)	0.478
Tumor size (>4 cm vs. ≤4 cm)	1.570 (1.140-2.164)	0.006	1.410 (1.008-1.973)	0.045
Tumor location	1.137 (0.956-1.351)	0.146	1.110 (0.917-1.318)	0.305
Differentiation status	1.388 (1.117-1.724)	0.003	1.328 (1.059-1.665)	0.014
CEA (elevated vs. normal)	1.194 (0.852-1.672)	0.303	1.260 (0.887-1.788)	0.197
CA199 (elevated vs. normal)	1.688 (1.213-2.350)	0.002	1.562 (1.103-2.211)	0.012
Depth of invasion	1.305 (1.154-1.477)	<0.0001	1.313 (1.152-1.496)	<0.0001
Lymph node metastasis	1.521 (1.335-1.732)	<0.0001	1.483 (1.293-1.700)	<0.0001
Metastasis	4.109 (2.603-6.486)	<0.0001	3.845 (2.411-6.132)	<0.0001
PSMD1 (high vs. low)	1.977 (1.428-2.739)	<0.0001	1.992 (1.417-2.801)	<0.0001

Table S3: Univariate association of PSMD1, clinicopathological characteristics with disease-free survival and overall survival in the validation cohort.

Variables	Disease-free survival		Overall survival	
	HR (95%CI)	p value	HR (95%CI)	p value
Age(years) (≥60 vs. <60)	1.391 (0.922-2.099)	0.116	1.338 (0.881-2.033)	0.172
Gender (female vs. male)	1.036 (0.687-1.564)	0.864	1.115 (0.733-1.696)	0.612
Tumor size (>4 cm vs. ≤4 cm)	1.518 (0.976-2.361)	0.064	1.523 (0.972-2.386)	0.066
Tumor location	1.174 (0.929-1.484)	0.179	1.181 (0.930-1.501)	0.172
Differentiation status	1.235 (0.908-1.681)	0.179	1.269 (0.926-1.740)	0.138
CEA (elevated vs. nomal)	1.690 (1.090-2.621)	0.019	1.771 (1.138-2.756)	0.011
CA199 (elevated vs. nomal)	1.419 (0.895-2.250)	0.136	1.521 (0.957-2.419)	0.076
Depth of invasion	1.348 (1.149-1.580)	0.0002	1.307 (1.118-1.528)	0.001
Lymph node metastasis	1.424 (1.209-1.678)	<0.0001	1.443 (1.222-1.705)	<0.0001
Metastasis	4.479 (2.503-8.015)	<0.0001	4.972 (2.778-8.898)	<0.0001
PSMD1 (high vs. low)	1.781 (1.171-2.708)	0.007	1.751 (1.144-2.682)	0.010
